# Pathogenic profiles and lower respiratory tract microbiota in severe pneumonia patients using metagenomic next-generation sequencing

**DOI:** 10.1007/s44307-025-00064-w

**Published:** 2025-04-25

**Authors:** Xinjie Han, Peng Ma, Chang Liu, Chen Yao, Yaxing Yi, Zhenshan Du, Pengfei Liu, Minlong Zhang, Jianqiao Xu, Xiaoyun Meng, Zidan Liu, Weijia Wang, Ruotong Ren, Lixin Xie, Xu Han, Kun Xiao

**Affiliations:** 1 College of Pulmonary & Critical Care Medicine, 8th Medical Center of Chinese PLA General Hospital, Beijing, China; 2 Chinese PLA Medical School, Beijing, China; 3 MatriDx Biotechnology Co., Ltd, Hangzhou, China; 4 School of Medicine, Nankai University, Tianjin, China; 5 Department of Urology, 8th Medical Center of Chinese PLA General Hospital, Beijing, China; 6 Foshan Branch, Institute of Biophysics, Chinese Academy of Sciences, Beijing, China

**Keywords:** CAP, HAP, mNGS, Pathogen Spectrum, Microbiota

## Abstract

**Introduction:**

The homeostatic balance of the lung microbiota is important for the maintenance of normal physiological function of the lung, but its role in pathological processes such as severe pneumonia is poorly understood.

**Methods:**

We screened 34 patients with community-acquired pneumonia (CAP) and 12 patients with hospital-acquired pneumonia (HAP), all of whom were admitted to the respiratory intensive care unit. Clinical samples, including bronchoalveolar lavage fluid (BALF), sputum, peripheral blood, and tissue specimens, were collected along with traditional microbiological test results, routine clinical test data, and clinical treatment information. The pathogenic spectrum of lower respiratory tract pathogens in critically ill respiratory patients was characterized through metagenomic next-generation sequencing (mNGS). Additionally, we analyzed the composition of the commensal microbiota and its correlation with clinical characteristics.

**Results:**

The sensitivity of the mNGS test for pathogens was 92.2% and the specificity 71.4% compared with the clinical diagnosis of the patients. Using mNGS, we detected more fungi and viruses in the lower respiratory tract of CAP-onset severe pneumonia patients, whereas bacterial species were predominant in HAP-onset patients. On the other hand, using mNGS data, commensal microorganisms such as *Fusobacterium yohimbe* were observed in the lower respiratory tract of patients with HAP rather than those with CAP, and most of these commensal microorganisms were associated with hospitalization or the staying time in ICU, and were significantly and positively correlated with the total length of stay.

**Conclusion:**

mNGS can be used to effectively identify pathogenic pathogens or lower respiratory microbiome associated with pulmonary infectious diseases, playing a crucial role in the early and accurate diagnosis of these conditions. Based on the findings of this study, it is possible that a novel set of biomarkers and predictive models could be developed in the future to efficiently identify the cause and prognosis of patients with severe pneumonia.

**Supplementary Information:**

The online version contains supplementary material available at 10.1007/s44307-025-00064-w.

## Introduction

As respiratory organs, healthy lungs facilitate the exchange of O2 and CO2 between the blood and the external environment, a process that is essential for maintaining the normal physiological functions of the human body. There has long been a lack of understanding of the microbiota of the healthy lung, and even a belief that the healthy lung (below the larynx) is sterile, due to less invasive sampling of the healthy lung and methodological limitations such as the ability to culture bacteria for complex biological samples(Moffatt and Cookson 2017). The widespread clinical use of high-throughput sequencing technologies in recent years has provided convenience and advantages to facilitate the identification and characterization of microbial populations in different parts of the human body(Integrative 2019).

Many studies have found a continuous and diverse microbiota in healthy lungs. Charlson ES et al. found *Candida* and *Aspergillus* in the BALF of healthy subjects(Charlson et al. 2012). Similarly, another observational study also found the presence of fungal biomes in healthy lungs(Martinsen et al. 2021). Hilty M et al. found the presence of bacteria including *Prevotella* spp and *Veillonella* spp in healthy lungs by 16S rRNA sequencing of cells obtained from left upper lobe bronchoscopic cytobrush sampling of healthy lungs(Hilty et al. 2010). In addition, viruses including rhinoviruses and phages have been found in the respiratory tract of healthy individuals(Thibeault et al. 2021). The pulmonary microbiota is in a continuous state of renewal, and its homeostasis plays a crucial role in regulating the immune response, inhibiting pathogen colonization, and maintaining host nutrient metabolism(Thibeault et al. 2021). Pulmonary microbial diversity is influenced by a variety of factors, including airway characteristics such as temperature, pH, antibiotic use, smoking, and a range of concomitant events associated with invasive mechanical ventilation (MV)(Martin-Loeches et al. 2020; Liu et al. 2022). Additionally, various lung diseases, including chronic obstructive pulmonary disease (COPD) (Xue et al. 2023), lung cancer (Tsay et al. 2021; Guo et al. 2023), tuberculosis (Dickson et al. 2014), cystic fibrosis (Lipuma 2010, 2014), and novel coronavirus pneumonia (Merenstein et al. 2021; Stavropoulou et al. 2020; Sulaiman et al. 2021), can exhibit varying degrees of microbial homeostasis. Dysbiosis of the lung microbiota is strongly associated with the progression and mortality of these diseases. However, the similarities and differences in microbiota across various diseases, as well as their clinical relevance, have not been extensively studied.

Lower respiratory infections (LRIs) remain the major global contributor to morbidity and mortality in 2021(GBD 2021a, 2021b). Pneumonia, a common clinical condition, is the most prevalent lower respiratory tract infection and poses a significant global health burden(Lanks et al. 2019; Jiang et al. 2024). Severe pneumonia, including CAP and HAP, its related mortality represents a significant cause of in-hospital death among patients(Microbial signatures predictive of short-term prognosis in severe pneumonia - PubMed n.d. 2025). CAP is an acute infection that causes 1.5 million hospitalizations each year in the United States(File and Ramirez 2023). HAP, the most frequent causes of hospital-acquired infection, is typically refractory and significantly affects the prognoses of critically ill patients(Yang et al. 2022). Diagnosing pathogens in severe pneumonia is challenging, making targeted treatment selection difficult. Timely and accurate determination of the etiology of pneumonia is imperative for initiating targeted therapeutic interventions effectively(Microbial signatures predictive of short-term prognosis in severe pneumonia - PubMed n.d. 2025). Nevertheless, conventional microbiological tests (CMTs) currently used often exhibit limitations in terms of sensitivity, speed, and the breadth of detectable pathogens. Recently, mNGS has become increasingly available and widely used in clinical practice(Chiu and Miller 2019; Miao et al. 2018; Chen et al. 2024; Hogan et al. 2021). mNGS allows for rapid etiological diagnoses of complex critical respiratory infections, covering a broader spectrum of pathogens (bacteria, viruses, fungi and parasites) in a single run when compared to routine tests(Li et al. 2020; He et al. 2022). The dynamics of the lung microbiota are linked to disease progression, and changes in the microbiota can influence patient outcomes and survival(Wolff et al. 2018). Previous studies have been carried out to verify the role of mNGS in both etiological diagnosis of pneumonia(Liu et al. 2024) and dynamics of lung microbiota(Jiang et al. 2024; Ao et al. 2023; Shen et al. 2023), especially in patients with CAP. However, only few studies have involved comprehensively microbial characterization and clinical features between HAP and CAP.

Thus, we conducted a retrospective study to evaluated the diagnostic performance of mNGS for identifying the composition and diversity of the lung microbiota in patients with suspected CAP and HAP. We also aimed to explore the featured microorganisms and analyse the clinical relevance of specific microbiota.

## Materials and methods

### Patient enrollment

The present study retrospectively enrolled patients with suspected pulmonary infection from January 2021 to April 2022 at the 8th Medical Center, Chinese PLA General Hospital. Totally, we screened 34 respiratory intensive care patients with CAP onset and 12 respiratory intensive care patients with HAP onset based on sample entry row criteria from 453 patients. All eligible patients were also divided into MV (Mechanical ventilation) and NMV (Non-mechanical ventilation) groups. Demographic information, clinical symptoms, laboratory test results, imaging examination results, diagnosis and treatment history, clinical course of the disease and outcomes were collected from electronic medical records.

This study was approved by the Ethics Committee of the 8th Medical Center, Chinese PLA General Hospital and conducted in accordance with Declaration of Helsinki principles and relevant ethical and legal requirements.

### Clinical sample collection and DNA extraction

BALF, sputum, peripheral blood and other samples were collected from each patient with the consent of themselves or their surrogates. The BALF samples were collected by experienced bronchoscopy specialists after the patient was anesthetized with midazolam. The peripheral blood was centrifuged at 1600 × *g* for 10 min and the collected supernatant was further centrifuged at 16,000 × *g* for 10 min to separate plasma. DNA extraction and library preparation from clinical samples were performed by using an NGS Automatic Library Preparation System (MatriDx Biotech Corp. Hangzhou). The quality of DNAs was assessed using a BioAnalyzer 2100 (Agilent Technologies; Santa Clara, CA, United States) combined with quantitative PCR to measure the adapters before sequencing.

### Metagenomic next-generation sequencing

Qualified DNA libraries were pooled together and subsequently sequenced on Illumina NextSeq500 system (50 bp single-end; San Diego, CA, United States). To control the quality of each sequencing run, a negative control and a positive control were conducted in parallel. A total of 10—20 million reads were generated for each sample. The raw sequenced reads were first processed with quality control to remove short (length < 35 bp), low quality and low complexity reads, as well as those corresponding to adapters. Host sequences were filtered out based on the alignment to the human-secific database in NCBI using Bowtie2 (version 2.3.5.1). The clean reads were thus aligned to a manual-curated microbial database using Kraken2 (version 2.1.2; confidence = 0.5) for quick taxonomic classification. The classified reads of interested microorganisms were further validated through a second alignment to the microbial database using Bowtie2. The classification of candidate reads might also be conducted by BLAST (version 2.9.0) whenever the results of Kraken2 and Bowtie2 were inconsistent.

Before data analysis, microbes detected in clinical samples were first compared with those detected in the parallel NTC (no template control). Microorganisms with a reads per million (RPM) above 10 or if the organism was not detected in the parallel NTC were maintained and defined as microbiota for further analysis. Substantially, all species of microbiota were looked up in PubMed to determine whether the organisms cause pneumonia and the positive pathogenic microorganisms were defined as pathogens.

### Statistical analysis

Categorical variables, shown as frequencies and percentages, were compared using Fisher’s exact test. Continuous measurement data following normal distribution were shown as mean ± standard deviation (x ± s), and non-normal distribution was shown by median (range). Differences and significance were calculated using the Wilcoxon test or Kruskal–Wallis test (for non-normal distribution data). SPSS 26.0 (IBM Corporation) was used for statistical analysis. Data visualisation was performed in R (Version 4.2.1). Specifically, bivariate or multivariate difference analysis was performed using unsupervised clustering methods with reference to the core steps of limma, voom, fit, eBays, etc. The final similarities (or differences) between samples were demonstrated using plotMDS() in the limma package and the results were output using the topTable method sorted by *P*-value. RPKM values of microbes were log2 transformed before their relative abundance was analysed. Variations in composition and abundance of microbes between groups were analyzed using the limma package. In this study, two-sided *P* values < 0.05 were considered statistically significant. Particularly, for multiple comparisons, FDR method was used to correct the primary *P*-value.

## Results

Assessment of the pathogenic spectrum in respiratory intensive care patients using mNGS and conventional microbiological assays.

**Fig. 1:**
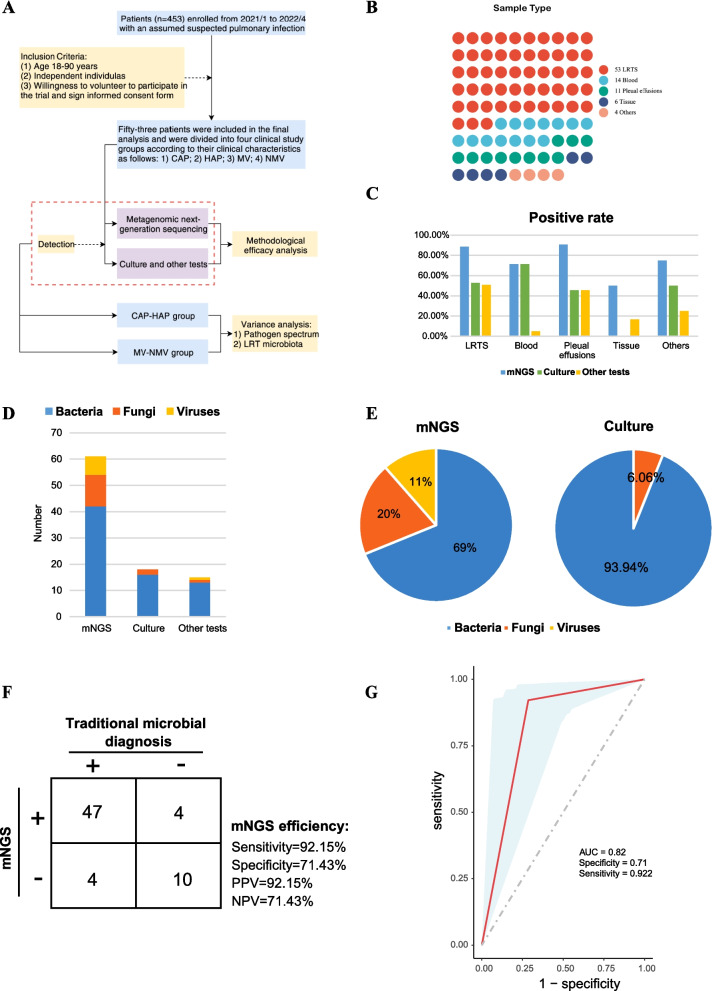
Study workflow and diagnostic performance assessment in the present study. (**A**-**B**) Flowchart and distribution of samples. (**C-E**) The positive rate of pathogens in different samples and types of detected pathogens based on mNGS, culture or other tests. (**F-G**) Performance assessment and receiver operating characteristic (ROC) curve of mNGS for pathogenic identification when compared with traditional microbial diagnosis

In this study, we screened 34 respiratory intensive care patients with CAP onset and 12 with HAP onset from 453 patients diagnosed with suspected pulmonary infections at the Department of Respiratory Intensive Care Medicine, PLA General Hospital VIII Medical Centre, between January 2021 and April 2022, based on sample inclusion and exclusion criteria (Fig. 1A, Table 1). The average length of hospital stay for the enrolled patients was 20.5 days (ranging from 2 to 48 days) and 28.6 days (ranging from 5 to 57 days), respectively (Table 1). Clinical samples such as BALF from the above patients were used for mNGS testing, routine microbiological testing, routine clinical test results and clinical consultation information collection. Compared to conventional pathogen detection methods (e.g. culture), mNGS can help detect more responsible pathogens, particularly viruses and fungi, in patients with respiratory critical illness (Fig. 1D-E & Figure S1). However, as shown in Table S1, there were still some pathogens detected by conventional methods but missed by mNGS. The results showed an overall pathogen detection rate of 71% using mNGS across all clinical samples (Figure S2A), with detection rates of 84% in LRTS, 72% in peripheral blood, 90% in pleural fluid, and 50% in tissue samples(Fig. 1C & Figure S2A). A total of 14 pathogens were identified by culture alone, whereas more pathogenic species were detected by mNGS test (Figure S2B-C). It was also observed that most of the clinical samples were mixed infections, with no more than 30% being single infection (Figure S2D-E). There were significant differences in the types of pathogenic bacteria detected in different clinical samples, with 68 predominating in BALF, 39 in peripheral blood, 28 in pleural fluid and 23 in tissue samples (Figure S1). Results showed that little overlap between the pathogens detected in peripheral blood, pleural effusion and tissue samples, whereas those detected in BALF overlapped more with those detected in peripheral blood and tissue, 43% and 27%, respectively (Fig. 1C-E & Figure S1). Using the final clinical diagnosis as an assessment criterion, we evaluated the performance of mNGS for the diagnosis of lung infection. It was demonstrated a sensitivity of 92.2% and a specificity of 71.4% for pathogen identification (Fig. 1F). The area under the curve (AUC) value of 0.82, as assessed by the clinical diagnostic results (Fig. 1G).

**Table 1:** Demographic and clinical characterics of CAP and HAP patients

Characteristic	CAP	HAP	*P*
**Age, mean (range), years**	65 (22–90)	66 (29–86)	0.848
**Sex, male, n (%)**	24 (68.57)	12 (80)	0.713
**White blood cell count, 10**^**9**^**/L**	13.20 (1.61–38)	12.13 (4.80–21.90)	0.619
< 4	1 (1.61)	0	NA
4–10	14 (4.7–10)	7 (4.8–10)	0.801
> 10	20 (10.23–38)	8 (12.1–21.9)	0.547
**Percentage of neutrophils**	84.20 (53.7–97.2)	85.05 (63.8–97.1)	0.709
40–75%	4 (53.7–71.9)	1 (63.8)	NA
> 75%	29 (75.2–97.2)	14 (76.6–97.1)	0.917
**Percentage of lymphocytes**	10.02 (1.3–39.8)	8.33 (1.3–13.3)	0.366
< 20%	31 (1.3–19.4)	15 (1.3–13.3)	0.79
20–50%	2 (21.8, 39.8)	0	NA
**CRP, mg/L**	106.98 (1.6–320)	119.86 (11.97–307.85)	0.579
< 10 mg/L	4 (1.6–9.7)	0	NA
10–50 mg/L	8 (20.3–40.92)	5 (11.97–33.4)	0.186
> 50 mg/L	21 (51.4–320)	10 (75.26–307.85)	0.704
**Procalcitonin, ng/mL**	1.72 (0.05–16.72)	6.67 (0.05–31.49)	0.031*
< 0.5 ng/mL	20/32	6/15	0.508
≥ 0.5 ng/mL	13/32	9/15	0.102
**Length of hospital stay, mean (range), days**	20.5 (2–48)	28.6 (5–57)	0.025*
**Antibiotic use, n (%)**	29 (82.86)	14 (93.33)	0.763

*NA* not applicable

### Characteristic pathogenic profiles and their clinical relevance in patients with severe CAP and HAP

**Fig. 2:**
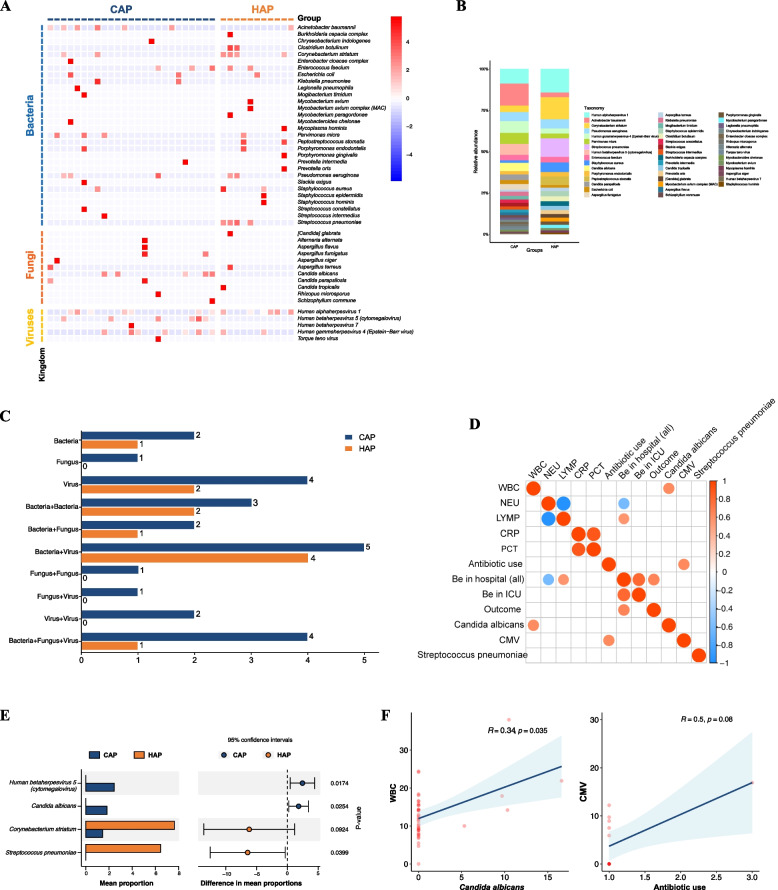
Pathogenic profiles of the lower respiratory tract and clinical relevance in patients with CAP-onset and HAP-onset identified by mNGS. (**A**) The heatmap of pathogens identified in patients with CAP-onset and HAP-onset. (**B**) Relative abundance of pathogens between patients with CAP-onset and those with HAP-onset. (**C**) Infectious patterns between patients with CAP-onset and those with HAP-onset. (**D**) Spearman correlations between the responsible pathogens and clinical characteristics. (**E**) Differential pathogens between patients with CAP-onset and those with HAP-onset. (**F**) Spearman correlation analysis between the key pathogens (*Candida albicans,* CMV) and clinical data (WBC, antibiotic use). Abbreviations: CMV, *Cytomegalovirus* (*Human betaherpesvirus 5*); WBC, White blood cells

The clinical samples used for mNGS identification involved BALF samples from 34 patients with CAP onset and 12 with HAP onset (Figure S3A). Among these patients, 11 were single infections and 27 were mixed infections (Figure S3B). mNGS methods detected over 60 pathogenic microbes, including bacterial (*n* = 45), fungal (*n* = 11) and viral species (*n* = 5) (Figure S3C). Significantly different pathogenic profiles of the lower respiratory tract were observed between respiratory critically ill patients with CAP onset and HAP onset, with more fungi and viruses detected in CAP patients and a predominance of bacteria detected in HAP patients (Fig. 2A-C). In terms of pathogen distribution characteristics, the frequency and relative abundance of bacterial pathogens were not significantly different between the CAP and HAP groups, but more fungi and viruses were detected in the CAP group, and their relative abundance was also higher (Fig. 2A & C). It was also found that *human herpesvirus type 5* (CMV) and *Candida albicans* were highly enriched in the CAP group, while *Corynebacterium striatum* and *Streptococcus pneumoniae* were highly enriched in the HAP group (Fig. 2B).

To explore the correlation between clinical characteristics and responsible pathogens in infectious severe pneumonia, we conducted a Spearman's test on the pathogens enriched in the CAP and HAP groups, examining their relationship with antibiotic use and length of stay. The results showed that white blood cell (WBC) was significantly and positively correlated with *Candida albicans* (r = 0.34, *P* = 0.035), suggesting that the WBC count in CAP patients may be used as an evaluation parameter for fungal infections (Fig. 2D-F). However, *cytomegalovirus* (CMV) did not show a significantly positive correlation with early antibiotic use in patients (r = 0.5, *P* = 0.08) (Fig. 2D-F).

### Comparative study of lower respiratory tract microbiota in critically ill patients with CAP and HAP, analysis of characteristic commensal microbial profiles and their clinical relevance

We compared the microbiota of BALF samples from different groups. A total of 605 microbial species were detected in all 42 BALF samples from CAP (*n* = 29) and HAP (*n* = 13) groups, the majority of which were bacteria (*n* = 550) and to a lesser extent fungi (*n* = 47), and viruses (*n* = 8) (Table S2). Figure S4A showed the overall microbial community for each sample, which provides comprehensive information on the distribution of microbiota in patients with severe pneumonia in the CAP and HAP groups. We generated heatmaps of the top 100 frequently occurring microbial species by log2 (RPKM + 1), including the top 70 bacteria, top 25 fungi and top 5 viruses (Figure S4A).

**Fig. 3:**
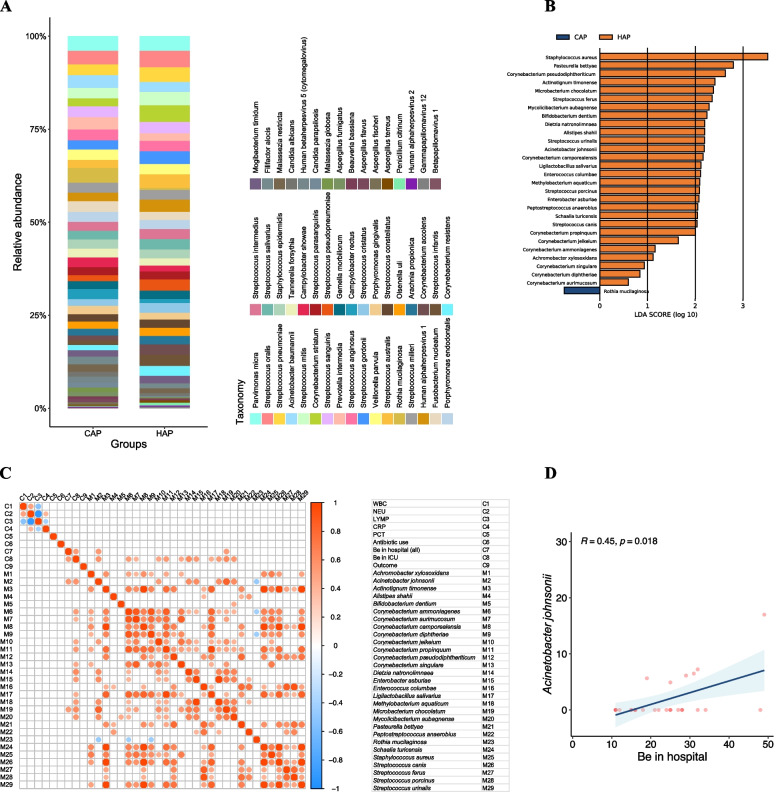
Microbial composition and clinical relevance in patients with CAP-onset and HAP-onset identified by mNGS. (**A**) Relative abundance of the top 50 microbial species between patients with CAP-onset and HAP-onset. (**B**) Comparison of microbial features between the two groups through the LEfSe analysis. (**C**) Spearman correlations between the differential microbes and clinical characteristics. (**D**) Spearman correlation between *Acinetobacte johnsonii* with clinical characteristic (hospital stay)

Figure 3 showed the microbial communities at the species level between the different groups. Figure 3A illustrated the differences in the relative abundance of the top 35 bacterial species, top 10 fungal species, and top 5 viral species detected in the BALF samples from severe pneumonia patients with CAP onset and HAP onset. The results showed that the species with overwhelming relative abundance were not consistent between the different groups. Although no significant differences in the general microbial diversity of the lower respiratory tract were observed between the CAP and HAP groups (Figure S4B-C), we still identified some highly enriched microorganisms in the HAP group and one highly enriched microorganism in the CAP group (Fig. 3B).

We wanted to know whether the different microbial composition was related to the clinical status of the patients. After screening for microorganisms with varying abundance in each group, we explored the correlation between clinical characteristics and these representative lung microorganisms. Spearman’s rank test was performed to investigate the associatuion of the key microbes enriched in the CAP and HAP groups described above with infection-related clinical indicators and clinical treatment parameters, i.e., antibiotic use and length of hospital stay (Fig. 3C). The results showed a significantly positive correlation between *Acinetobacter johnsonii* and length of hospital stay (r = 0.45, *P* = 0.018) (Fig. 3D).

### Characteristic pathogenic and commensal microbes and their clinical relevance in MV and NMV patients

All eligible patients were also divided into MV patients (*n* = 22) and NMV patients (*n* = 24) based on whether or not they received mechanical ventilation (Figure S5A, Table 2). Different types of the pathogens and different kinds of infections were also detected among the present patients (Figure S5B), but no significant difference was observed between patients in MV and NMV groups as shown in the Figure S5C-E. There were also no differences in the general microbial diversity of the lower respiratory tract between patients from MV and NMV groups (Figure S6A-C). Nevertheless, LEfSe analysis revealed several commensal microorganisms such as *staphylococcus epidermidis* enriched in MV patients rather than in NMV patients (Figure S6D). The clinical correlation results visualized by heatmap showed that the percentage of neutrophil (NEU%) was negatively correlated with *Acinetobacter bereziniae* (r = -0.41, *P* = 0.044) and *Kocuria palustris* (r = -0.53, *P* = 0.013) (Figure S6E).

**Table 2:** Demographic and clinical characterics of MV and NMV patients

Characteristics	MV	NMV	*P*
**Age, mean (range), years**	66 (22–90)	63.81 (29–81)	0.595
**Sex, male, n (%)**	17 (73.91)	20 (64.52)	0.521
**White blood cell count, 10**^**9**^**/L**	14.77 (1–38)	10.37 (1.61–33)	0.024*
< 4	1 (1)	1 (1.61)	
4–10	4 (4.8–10)	18 (4.6–10)	0.523
> 10	16 (12–38)	11(10.23–33)	0.404
**Percentage of neutrophils**	85.76 (63.8–95.2)	82.16 (53.7–91.4)	0.126
40–75%	1 (63.8)	5 (53.7–74.3)	
> 75%	20 (78.5–95.2)	25 (76.6–91.4)	0.252
**Percentage of lymphocytes**	8.47 (1.3–18.4)	11.52 (4.3–39.8)	0.099
< 20%	21 (1.3–18.4)	28 (4.3–19.4)	0.349
20–50%	0	2 (25.4, 39.8)	
**CRP, mg/L**	106.12 (11.97–304)	99.33 (1.5–320)	0.878
< 10 mg/L	0	5 (1.5–9.7)	
10–50 mg/L	8 (11.97–40.92)	6 (6.83–37.11)	0.372
> 50 mg/L	13 (51.4–304)	19 (54.51–320)	0.838
**Procalcitonin, ng/mL**	2.61 (0.05–31.49)	3.23 (0.05–36.3)	0.762
< 0.5 ng/mL	10/20	20/31	0.056
≥ 0.5 ng/mL	10/20	11/31	0.293
**Length of hospital stay, mean (range), days**	22.4 (2–49)	21.6 (1–57)	0.828
**Antibiotic use, n (%)**	19 (82.60)	23 (74.19)	0.346

*NA* not applicable

## Discussion

In this study, we performed a comprehensive statistical analysis of the clinical features of patients with suspected infectious severe pneumonia, who were divided into different groups as CAP/HAP and MV/NMV. The current study found that different pathogens and infection patterns (single and mixed infections) result in various non-specific clinical features, complicating the identification of the causative pathogen in infectious severe pneumonia(Lanks et al. 2019; Shao et al. 2024; Liu et al. 2023). As a high-throughput sequencing-based assay, mNGS has shown good diagnostic performance in identifying a variety of pathogens, such as bacteria, fungi, viruses and parasites, which is valuable for rapid and accurate diagnosis of mixed infections(Liang et al. 2022). Our study showed that mNGS was able to clearly distinguish infections with different pathogenic species and identify additional co-infecting pathogenic microorganisms.

The respiratory microbiota contains specific ecological niches of commensal and pathogenic microorganisms that plays an important role in determining the onset and progression of disease(Andrade et al. 2022; Man et al. 2017; Dickson et al. 2016). The diversity of microorganisms in the lungs may be influenced by a variety of biotic or abiotic conditions(Liu et al. 2022). Imbalances in the respiratory microbiota may be associated with increased colonization by opportunistic pathogens, leading to severe pneumonia(Thibeault et al. xxxx; Pascale et al. 2021; Narendrakumar and Ray 2022). Changes in the microbiota have been observed during lower respiratory tract infections and are strongly associated with the progression and prognosis of pneumonia(Hanada et al. 2018). Therefore, a better understanding of changes in microbiota composition would go a long way in elucidating the role of pathogens in lung infections. Several studies have shed light on the lung microbiota of children with bacterial meningitis(Liao et al. 2021), refractory Mycoplasma pneumonia(Zhou et al. 2022), tuberculosis(Xiao et al. 2022; Ding et al. 2021) and invasive pulmonary aspergillosis(Hérivaux et al. 2020) through untargeted pathogen metagenomics or 16S rRNA gene sequencing. Although 16S rRNA gene sequencing can annotate bacteria up to intermediate genus-level and species-level identification, it does not provide comprehensive whole-microbe information and higher resolution of metagenomic technologies (e.g. shotgun sequencing), especially for closely related species(Boers et al. 2019).

In this study, we investigated the lung microbial communities of patients with different types of severe pneumonia by mNGS sequencing. Currently, BALF samples are widely used for pathogen detection in lower respiratory tract infections, as they exhibit minimal oral microbial contamination and provide accurate, representative information on lung microbiota(Bingula et al. 2020; Fenn et al. 2022; Jin et al. 2022; Zhang et al. 2022). The lung microbiota study conducted in this study explored BALF samples from patients with severe pneumonia with CAP onset and HAP onset. Because the sample grouping was derived from a clear clinical diagnosis and retrospective origin of the onset, data problems due to sample bias were largely avoided. Our results showed that the microbiota profiles in BALF samples from patients with severe pneumonia with CAP onset and HAP onset did not show significant differences in alpha diversity and abundance, which is different from previous observations in patients with ventilator-associated pneumonia(Fenn et al. 2022). A valuable study reported that patients diagnosed with invasive pulmonary aspergillosis showed a reduction in alpha diversity driven by altered abundance of bacterial genera(Hérivaux et al. 2022). A reduction in microbial diversity can make the lower respiratory tract more susceptible to colonization by dominant pathogens(Natalini et al. 2023).

Previous studies have found that changes in various clinical indicators in patients with *Pneumocystis carinii* pneumonia were closely associated with changes in the abundance of respiratory microbiota(Samuelson et al. 2016). As significant differences between groups were observed in our study in terms of some clinical features, key responsible pathogens and representative microbial species, we sought to correlate these findings to further explore the potential role of microbes in patients' conditions. Notably, we identified a range of microorganisms that were highly enriched in samples from patients with HAP-initiating severe pneumonia, while total patient length of stay and ICU stay increased significantly with increasing relative abundance of these microorganisms. We hypothesize that the impact of pathogens on lung microorganisms ultimately determines the physical status of patients and the progression of disease. This study provides evidence linking the causes of infection, pathogenic agents, modes of infection, pulmonary microbial changes, and clinical parameters, offering insights into the pathogenesis of severe infectious respiratory diseases.

We should note that our study has several limitations. First, the small patient population in our study did not allow for a large sample size polymorphism analysis to characterize differences in the lung microbiome of different subgroups. More broadly, future studies will require larger samples to draw more definitive conclusions about the microbial diversity of the lower respiratory tract in patients with severe pneumonia. Secondly, although patients with severe pneumonia of different origins exhibit variations in their lower respiratory microbiota, it remains unclear how these variations impact the long-term prognosis and whether differences in the microbiota persist after patients have recovered. Some studies have reported that microbial imbalances can induce systemic metabolic alterations(Nieuwdorp et al. 2014; Devaraj et al. 2013) and host immune responses(Shi et al. 2017; Xia et al. 2023), so the functional characterization of the lung microbiota and the correlation between microbial and host immunity should be further explored and characterized.

## Supplementary Information

Additional file 1: Figure S1. Pathogens detected in all clinical samples (LRTS, peripheral blood, pleural fluid and tissue) based on mNGS, culture and other tests. The bar on the right represents the total frequency of each pathogenic species detected in all samples by mNGS, culture and other tests, and the percentages represents the total positive rate of each pathogen. The bar on the top represents the accumulated frequency of pathogenic species detected in each sample by mNGS, culture and other tests.

Additional file 2: Figure S2. Pathogens detected in all clinical samples based on mNGS test, and culture. (A) Positive rate of mNGS test in different samples and in all samples. (B) Pathogens identified by culture (all of pathogens). (C) The top 20 of pathogens identified by mNGS test. (D) Infectious patterns in all clinical samples. (E) Proportion of single and mixed infection in all clinical samples.

Additional file 3: Figure S3. Pathogens identified in samples from CAP-onset patients and HAP-onset patients through mNGS, culture and other tests. (A) The mumber of CAP-onset patients and HAP-onset patients. (B) Proportion of single and mixed infections among the current patients. (C) The overall profiles of pathogens identified in patients with CAP-onset and HAP-onset through mNGS, culture and other tests.

Additional file 4: Figure S4. Microbial diversity of the lower respiratory tract between CAP-onset and HAP-onset patients. (A) Heatmap of the top 100 frequently occurring microbial species. (B) Microbial alpha-diversity analysis based on Shannon, Simpson and inverse Simpson indices. (C) Microbial beta-diversity analysis.

Additional file 5: Figure S5. Pathogens identified in patients from MV and NMV groups through mNGS, culture and other tests. (A) The number of MV patients and NMV patients. (B) Different types of the pathogens detected and different infection patterns among the present patients. (C) The overall spectrum of pathogens identified in patients from MV and NMV groups through mNGS, culture and other tests. (D) Relative abundance of the pathogenic species between MV and NMV groups. (E) Differential analysis on the pathogenic species between MV group and NMV group.

Additional file 6: Figure S6. Microbial diversity of the lower respiratory tract between MV and NMV patients. (A) Heatmap of the top 100 frequently occurring microbial species. (B) Microbial alpha-diversity analysis based on Shannon, Simpson and inverse Simpson indices. (C) Microbial beta-diversity analysis. (D) Comparison of microbial features between the two groups through the LEfSe analysis. (E) Spearman correlations between the differential microbes and clinical characteristics.

Additional file 7. Table S1. Pathogens detected by conventional methods but missed by mNGS.

Additional file 8.

## Data Availability

The datasets presented in this study can be found in NCBI with the SRA accession PRJNA942361.

## Abbreviations

mNGS: Metagenomic next-generation sequencing
CAP: Community-acquired pneumonia
HAP: Hospital-acquired pneumonia
MV: Mechanical ventilation
NMV: Non-mechanical ventilation
LRT: Lower respiratory tract
LRTS: Lower respiratory tract samples
BALF: Bronchoalveolar lavage fluid
ROC curve: Receiver operating characteristic curve
AUC: Area under the curve
EBV: Epstein-Barr virus (Human gammaherpesvirus 4)
CMV: Cytomegalovirus (Human betaherpesvirus 5)
NTC: No template control
RPM: Reads per million
LEfSe: Linear discriminant analysis effect size
LDA: Linear discriminant analysis
PCoA: Principal coordinate analysis
WBC: White blood cells
NEU: Neutrophil
LYMP: Lymphocyte ratio
CRP: C-reactive protein
PCT: Procalcitonin
