# Supplementary figures and images for: Pathogenic profiles and lower respiratory tract microbiota in severe pneumonia patients using metagenomic next-generation sequencing
Source: Adv Biotechnol (Singap). 2025 Apr 25;3(2):13. doi: 10.1007/s44307-025-00064-w (PMC12031718; doi:10.1007/s44307-025-00064-w)

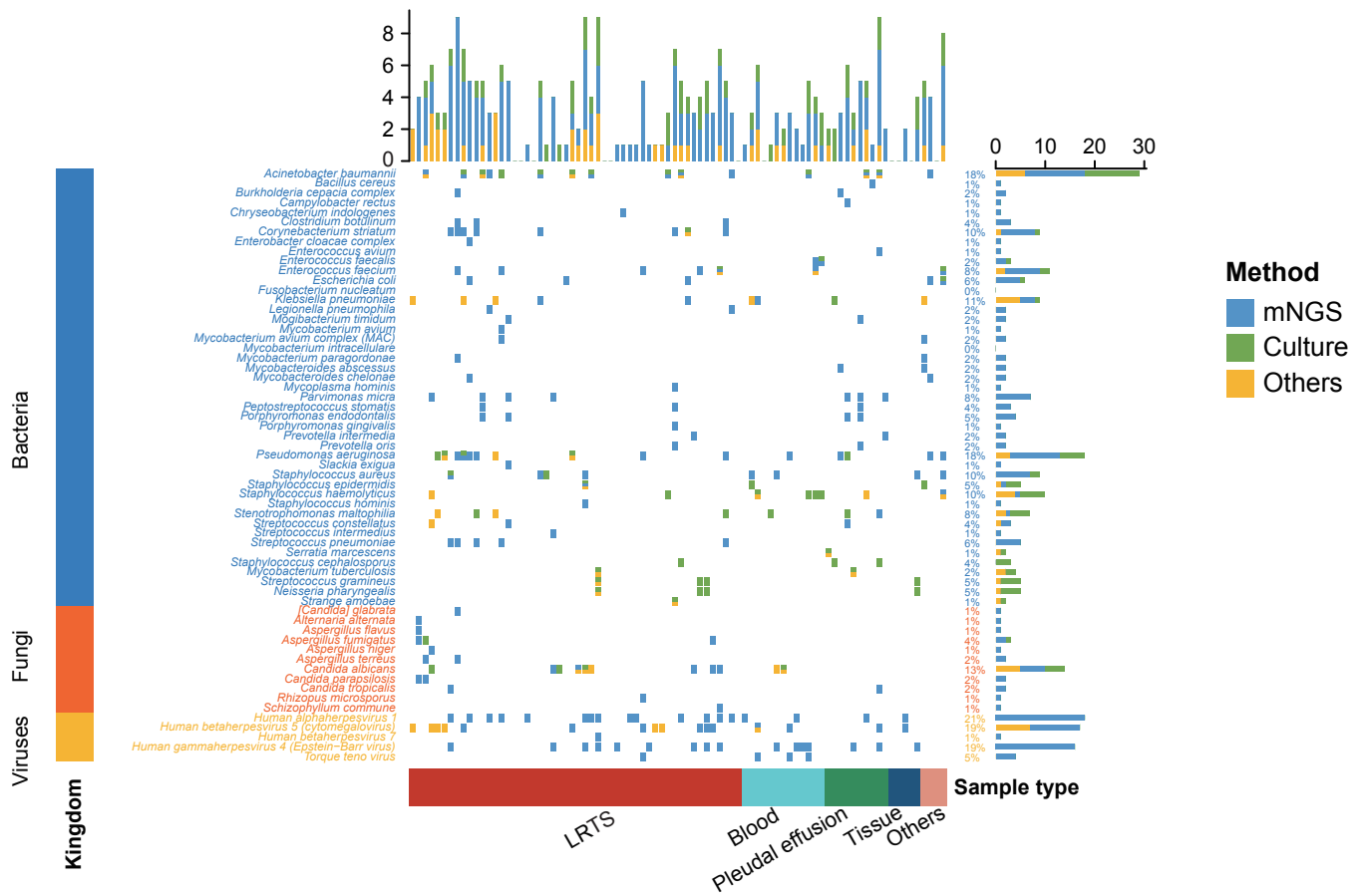

Supplement: Supplementary file 1 — Additional file 1: Figure S1. Pathogens detected in all clinical samples (LRTS, peripheral blood, pleural fluid and tissue) based on mNGS, culture and other tests. The bar on the right represents the total frequency of each pathogenic species detected in all samples by mNGS, culture and other tests, and the percentages represents the total positive rate of each pathogen. The bar on the top represents the accumulated frequency of pathogenic species detected in each sample by mNGS, culture and other tests. [file 44307_2025_64_MOESM1_ESM.pdf]

A

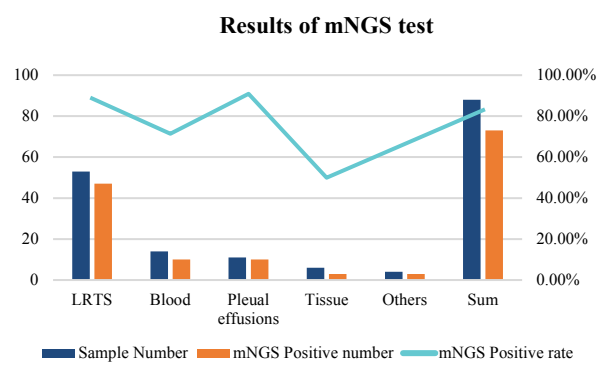

B

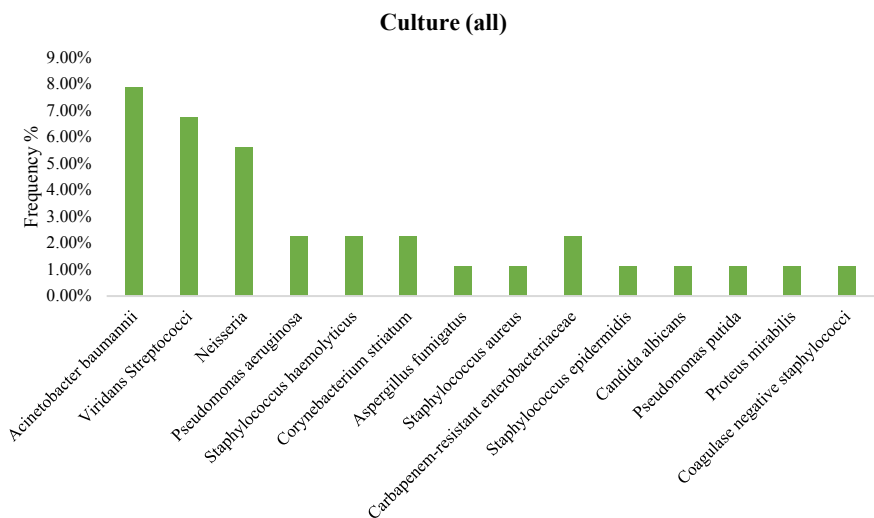

C

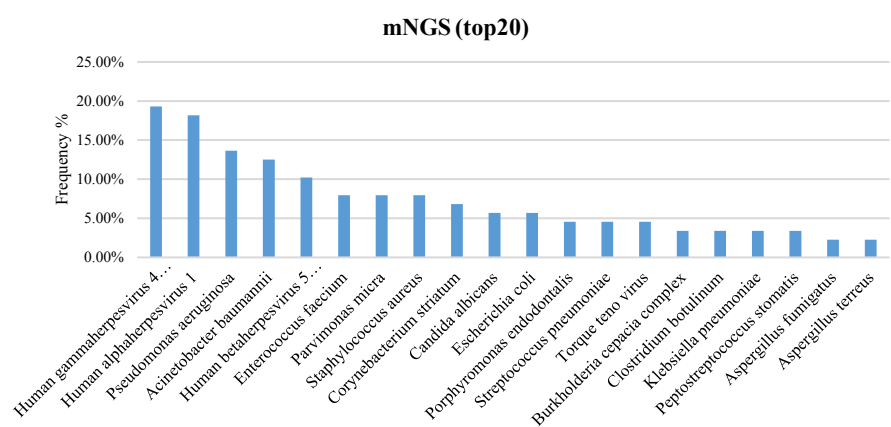

D

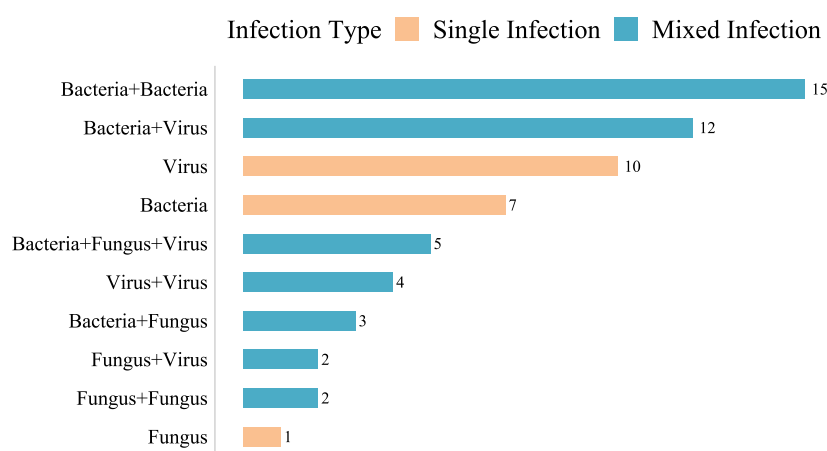

E

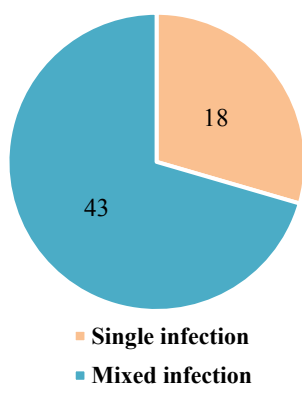

Supplement: Supplementary file 2 — Additional file 2: Figure S2. Pathogens detected in all clinical samples based on mNGS test, and culture. (A) Positive rate of mNGS test in different samples and in all samples. (B) Pathogens identified by culture (all of pathogens). (C) The top 20 of pathogens identified by mNGS test. (D) Infectious patterns in all clinical samples. (E) Proportion of single and mixed infection in all clinical samples. [file 44307_2025_64_MOESM2_ESM.pdf]

A

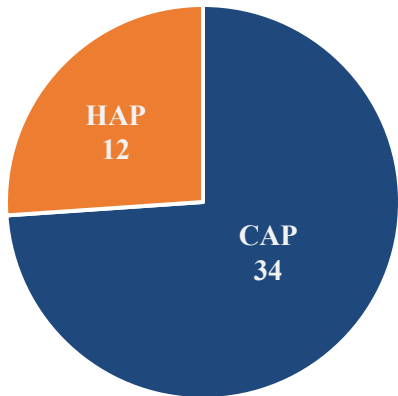

B

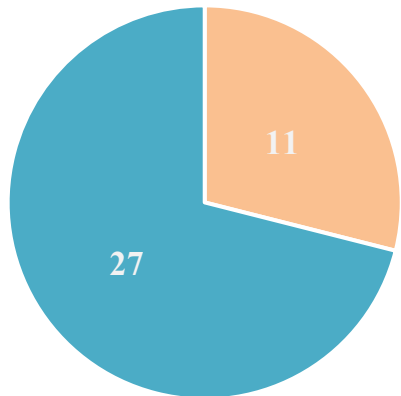

Single infection  
Mixed infection

C

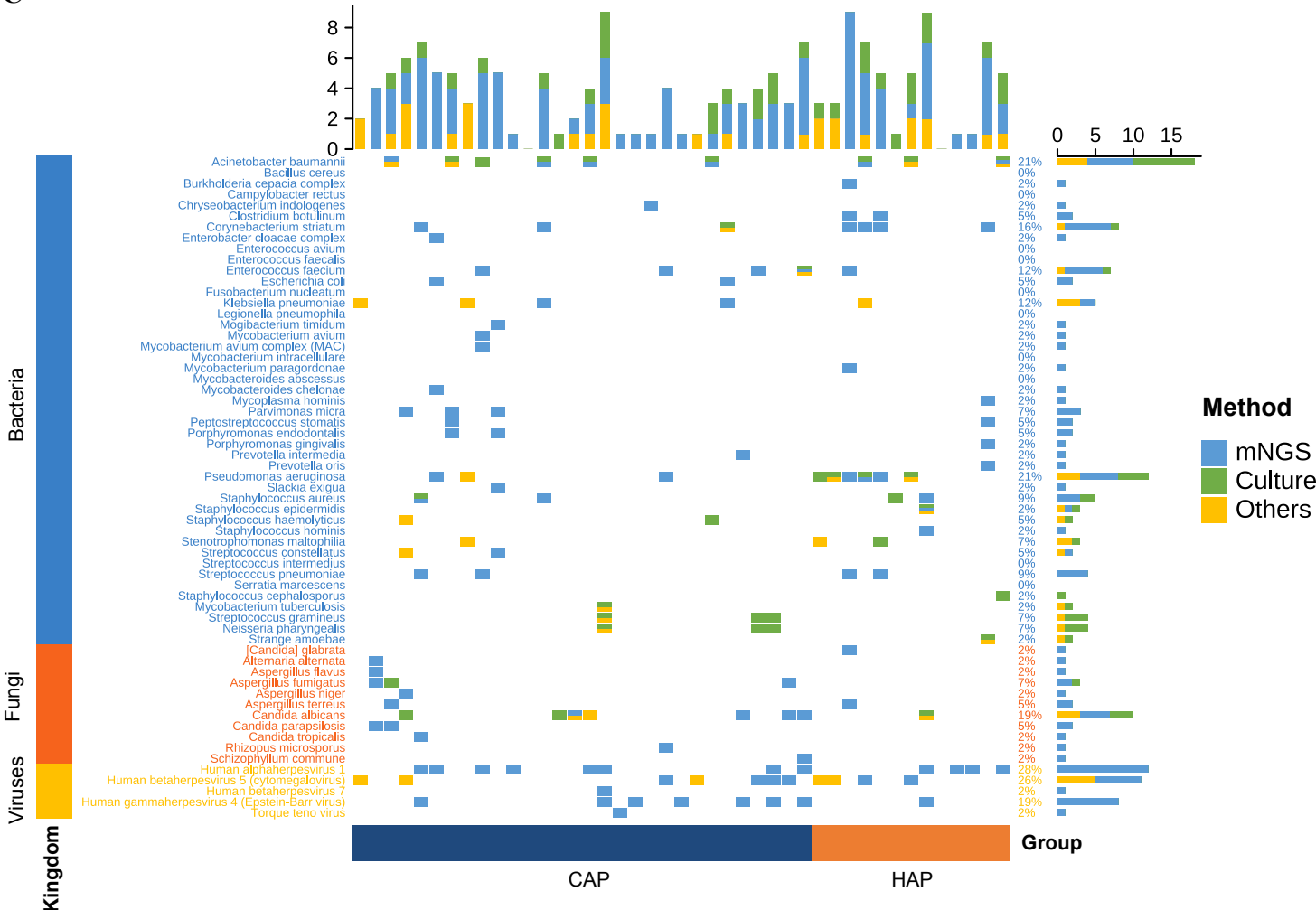

Supplement: Supplementary file 3 — Additional file 3: Figure S3. Pathogens identified in samples from CAP-onset patients and HAP-onset patients through mNGS, culture and other tests. (A) The mumber of CAP-onset patients and HAP-onset patients. (B) Proportion of single and mixed infections among the current patients. (C) The overall profiles of pathogens identified in patients with CAP-onset and HAP-onset through mNGS, culture and other tests. [file 44307_2025_64_MOESM3_ESM.pdf]

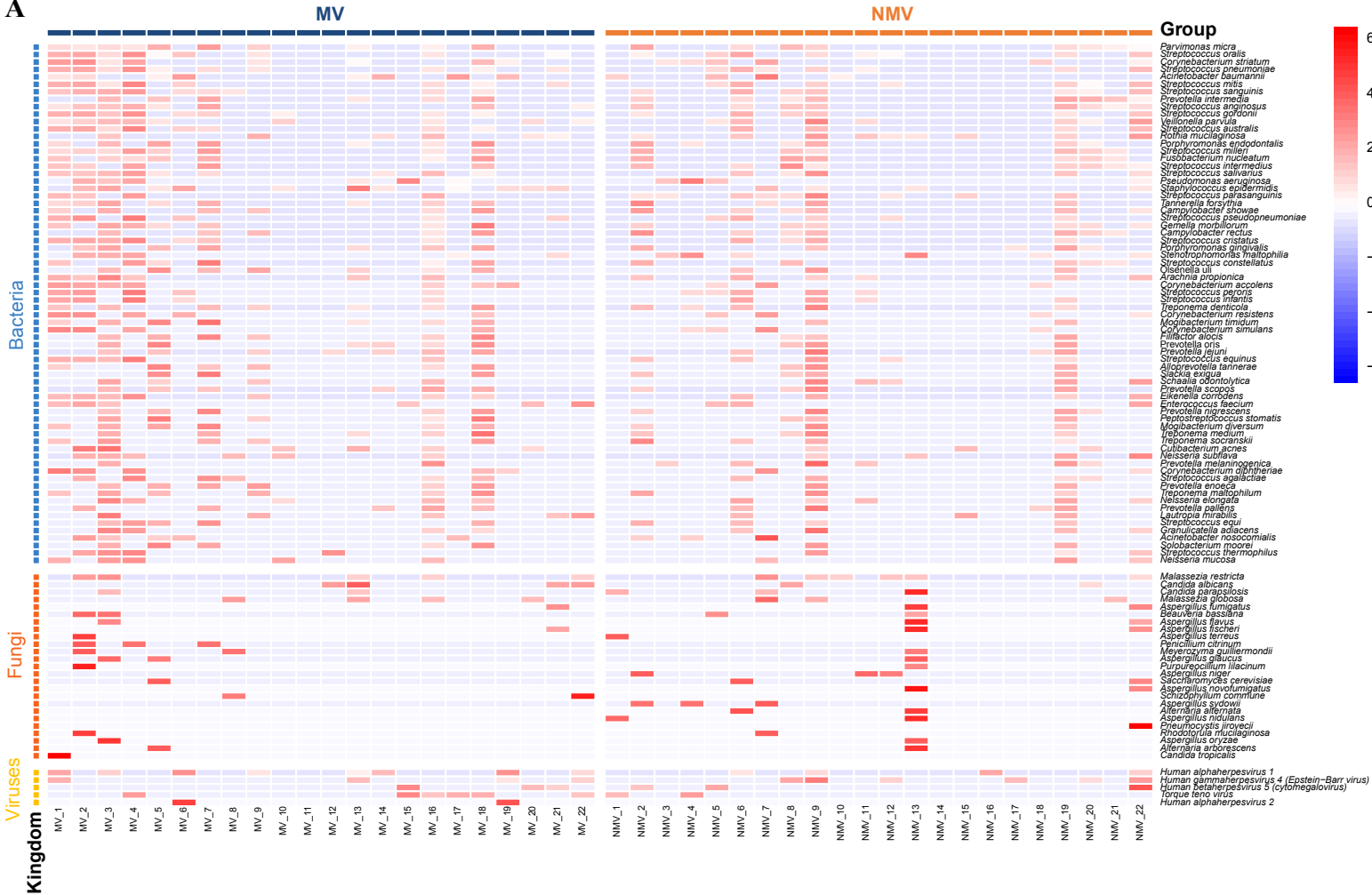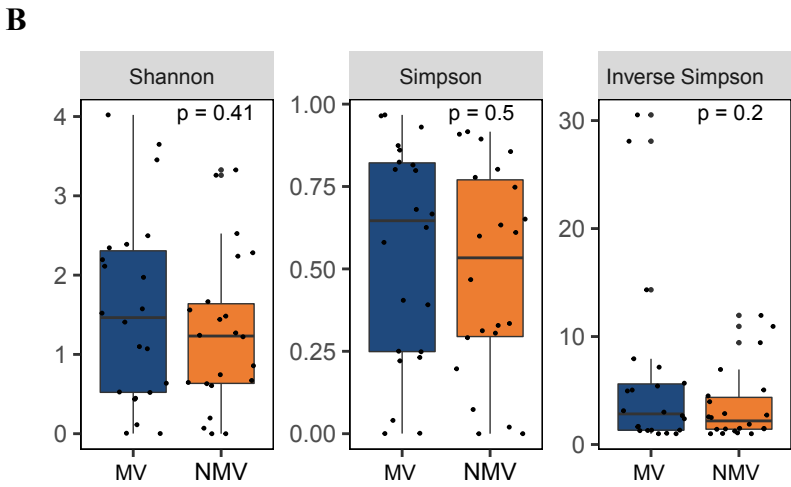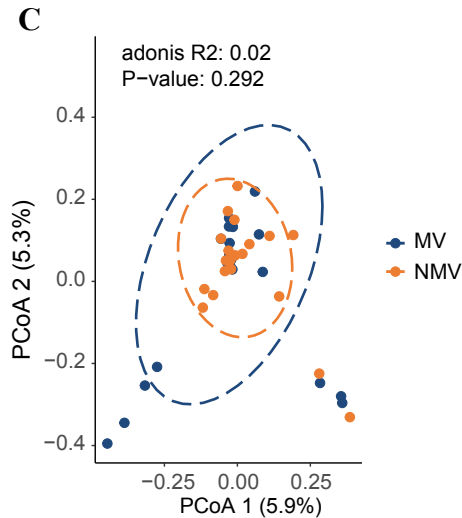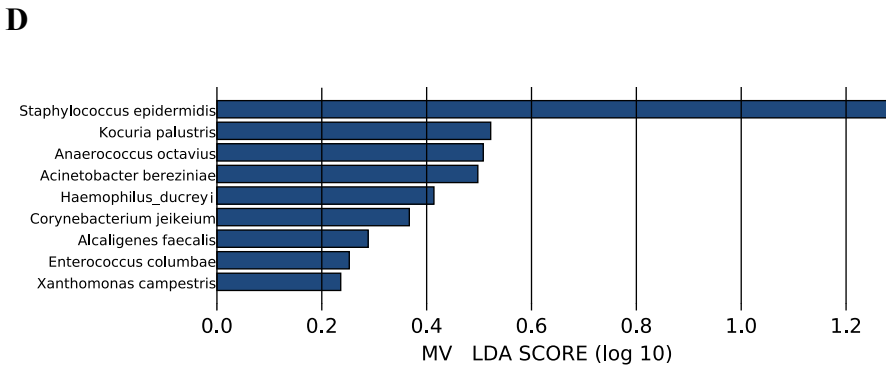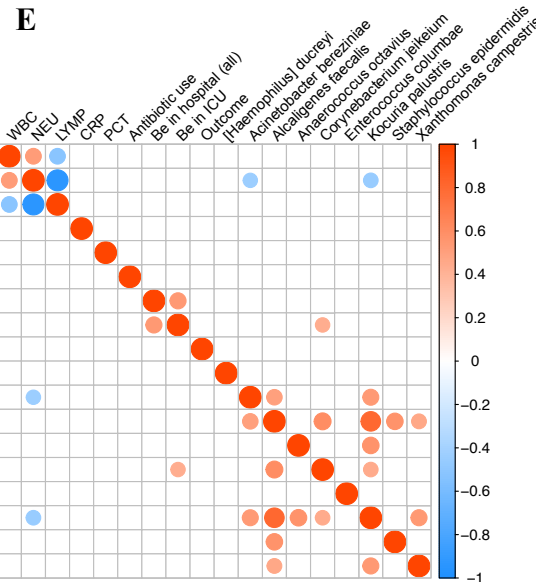

Supplement: Supplementary file 4 — Additional file 4: Figure S4. Microbial diversity of the lower respiratory tract between CAP-onset and HAP-onset patients. (A) Heatmap of the top 100 frequently occurring microbial species. (B) Microbial alpha-diversity analysis based on Shannon, Simpson and inverse Simpson indices. (C) Microbial beta-diversity analysis. [file 44307_2025_64_MOESM4_ESM.pdf]

A

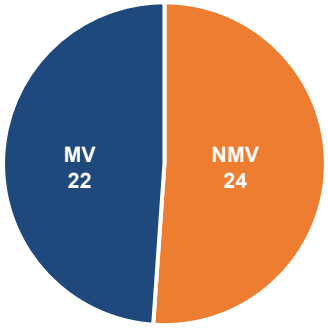

B

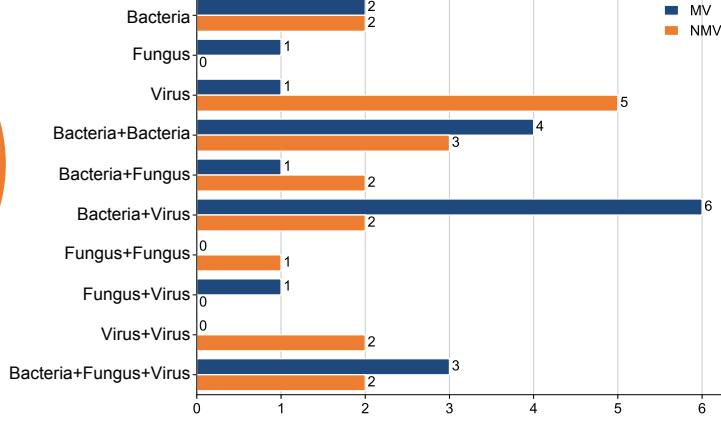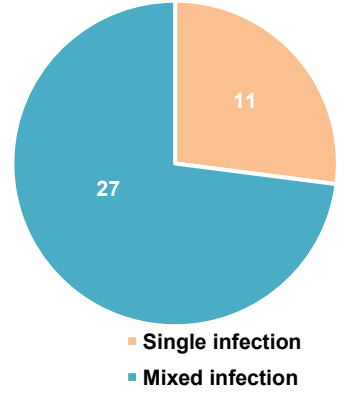

C

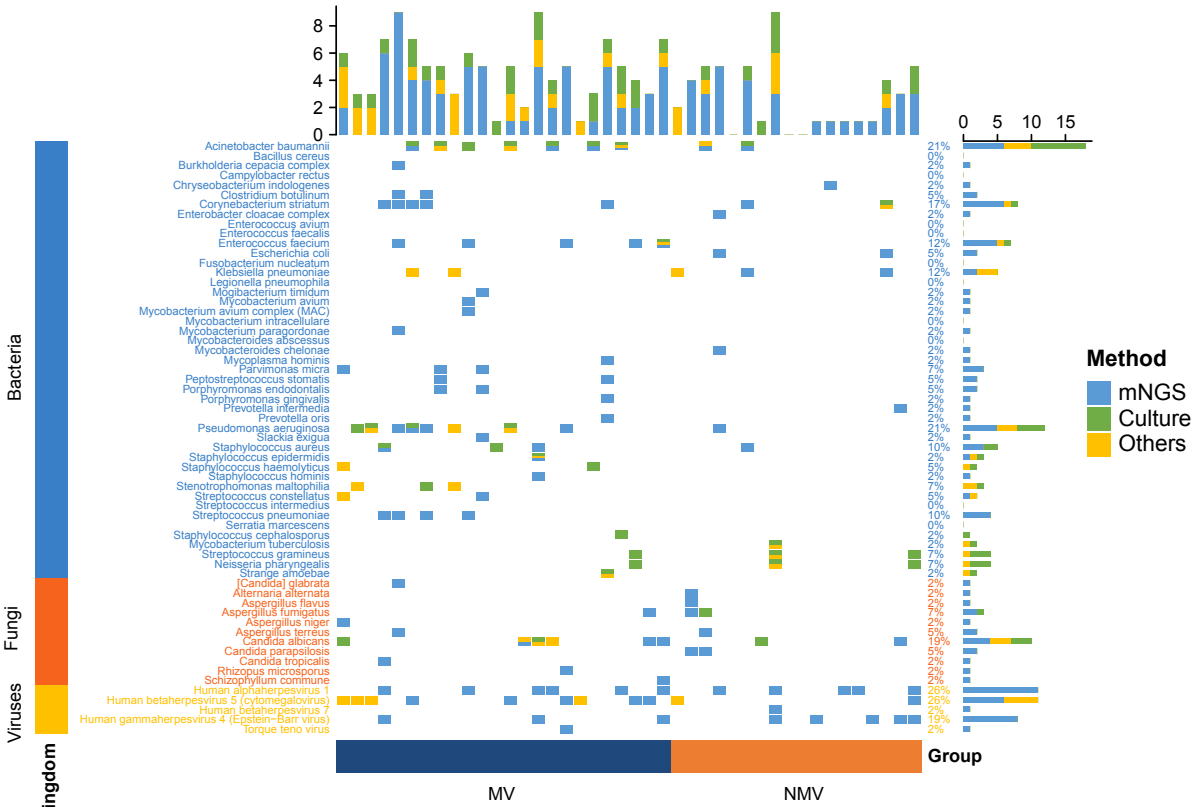

D

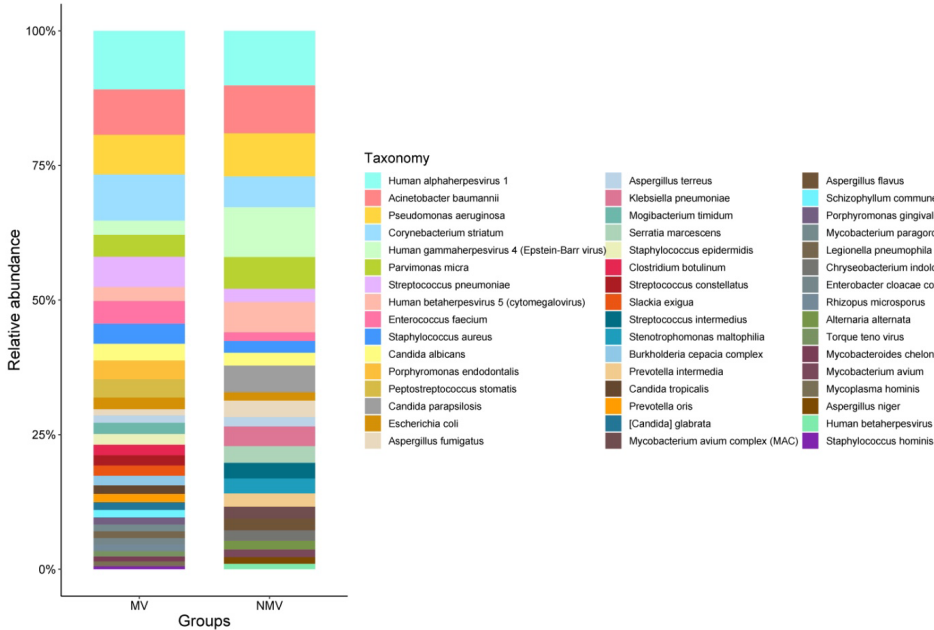

E

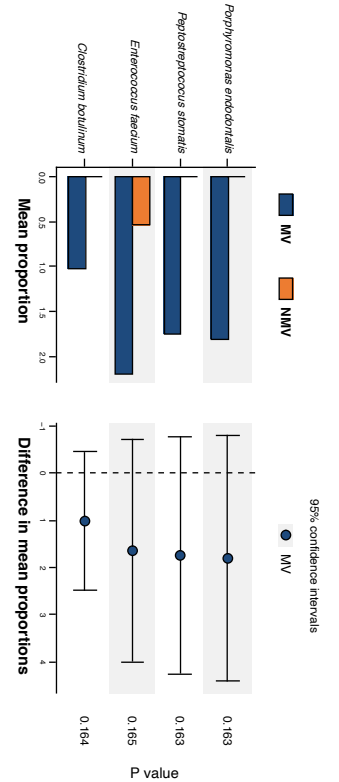

Supplement: Supplementary file 5 — Additional file 5: Figure S5. Pathogens identified in patients from MV and NMV groups through mNGS, culture and other tests. (A) The number of MV patients and NMV patients. (B) Different types of the pathogens detected and different infection patterns among the present patients. (C) The overall spectrum of pathogens identified in patients from MV and NMV groups through mNGS, culture and other tests. (D) Relative abundance of the pathogenic species between MV and NMV groups. (E) Differential analysis on the pathogenic species between MV group and NMV group. [file 44307_2025_64_MOESM5_ESM.pdf]
